# Attributes of Interactive Online Health Information Systems

**DOI:** 10.2196/jmir.7.3.e33

**Published:** 2005-07-01

**Authors:** Joseph B Walther, Suzanne Pingree, Robert P Hawkins, David B Buller

**Affiliations:** ^4^Klein Buendel IncGoldenCOUSA; ^3^Department of Journalism and Mass CommunicationUniversity of WisconsinMadisonWIUSA; ^2^CHESS ProjectUniversity of WisconsinMadisonWIUSA; ^1^Department of CommunicationCornell UniversityIthacaNYUSA

**Keywords:** Cancer support, interactivity, presence, homophily, social support, sociotechnical factors

## Abstract

The development of online communication systems related to prevention, decision making, and coping with cancer has outpaced theoretical attention to the attributes that appeal to system users and that create effective interactions. This essay reviews a number of sociotechnical attributes related to online discussion systems and tutorials, including interactivity, presence, homophily, social distance, anonymity/privacy, and interaction management. These attributes are derived from different theoretical perspectives which have led to clinical trials and other empirical studies demonstrating effectiveness or attraction to end users. The effects of a subset of these attributes are connected to learning, social influence, and coping, as illustrated in evaluations of an interactive smoking prevention site and a cancer advice/support discussion system.

## Introduction

The Internet has become a beacon of information and support to many patients, caregivers, and survivors of cancer. Numerous statistics show the popularity of the Internet among this population, numerous efforts continue to grow in the purposeful development and refinement of online services for these individuals, and numerous groups continue to expand and refine their own self-organized, informal online discussion and chat systems to help support information exchange and coping. Despite their potential, online health systems have only recently become the topic of scientific investigation with healthy, but at-risk, populations in community settings. Studies on programs intended to teach healthy eating habits [1-4], promote healthy body images [5-8], manage weight [9,10], promote tobacco cessation [11,12], and increase physical activity [4] have been reported. Some of these programs merely provided online information, while a few attempted to capitalize on the medium's interactivity to deliver content tailored to the user. The results are mixed, at present, with some studies finding benefits from Internet programs [3,5,7,10] and others not [1,8].

While efforts in all these directions are inspiring and encouraging, the advancement of practical efforts requires theoretical understanding of the potentially unique and variable attributes that online information systems and peer discussion systems offer for their users. By understanding what works in native and purposive Internet environments, we can identify those elements that offer the most promise and effectiveness for the specific design of Internet-based systems to enhance and facilitate cancer patients' health and well-being. This review will focus on several attributes of social technology that have been identified in online support groups and online information systems. They include interactivity, presence, social network attributes (expertise and distance), homophily, anonymity, and interaction management. Not all of these attributes are most pertinent in every type of Internet health support system, but each holds promise for the relative attractiveness and effectiveness of different Internet health information venues. The relationships of some of these variables—especially interactivity and presence—are linked through learning, social influence, or other moderating perceptions to attitudinal and potential behavioral responses related to cancer prevention, decision making, and coping. Results of previous studies and ongoing development illustrate some of these relationships and suggest hypotheses for additional understanding and future directions for system development.

## Attributes

### Interactivity

Interactivity has been called a defining feature of online technologies, with a particular focus on tailoring content to users, increasing engagement in decision making, improving learning, increasing attractiveness, and enhancing the influence of online services [13]. Most definitions require an exchange of information, responsiveness, and some variation on user control.

Human communication processes and outcomes vary systematically with the degree of interactivity—some form of interdependent exchange—in a communication modality [14-17]. Interactivity includes structural principles of contingency (tailored responses to user queries), participation (active rather than passive user behavior), synchronicity (real time rather than delayed exchange), proximity (in the geographical sense), and richness of nonverbal contextual information. Experientially, it includes individual involvement (cognitive, sensory, visceral), mutuality (interdependence, shared understanding), and individuation (well-defined actors). With database functions and dynamic Web page technology, online health information systems can collect information from users and adapt content to them immediately, in real time and at any time (contingent and synchronous) [18]. Interfaces can be programmed to permit self-navigation (user involvement) among databases and multimedia programs using seamless hypertext links [19-21], without resorting to complicated, expensive expert systems. Chat room, bulletin board, and email technologies can deliver prevention messages to users, and online counseling can heighten the sense of mutuality and individuation [22,23].

### Presence

Current explications of presence [24] make several key distinctions worth repeating here. First, presence is not defined either by technology or by the situation the person is in; instead, presence is a human perceptual response subjectively created by an interaction of situation, technology, and individual needs and expectations. Second, these explications distinguish between physical, social, and self domains for the experience of presence and then cross these domains with the distinction between whether the object experienced is real, but not present, or is only virtual. Thus, computer-stimulated physical presence occurs when the user subjectively experiences non-present real or virtual objects. Social presence involves perceived contact with real or imaginary others. And self presence occurs when the computer interaction produces revelations or alterations of self-perception.

In line with the definitions above, it is important to note that presence, like interactivity, does not depend on real-time message exchange. While real-time, or synchronous, interaction is appealing to some users some of the time, asynchronous technologies have a valuable place in cancer support. Indeed, the manner in which online message storage systems arrange postings by topical “thread” and archive messages for opportunistic browsing by users wherever and whenever they have the time to find them does not diminish the level of emotion or perceived reality of the shared experiences of participants.

Of these, physical presence may be irrelevant to typical cancer patients' experiences with interactive cancer communication systems. (Some video games, mainly aimed at children, involve blasting cancer cells and could conceivably offer some sense of physical presence and efficacy.) Whether or not online discussion systems or expert advice systems stimulate physical, or merely virtual, presence seems unclear at this point, and perhaps it is theoretically meaningless. However, we argue that social presence, both with real and virtual others, is important and consequential for cancer patients.

Lee [24] has proposed that interactivity may be a necessary condition for presence. That is, a system over which a user has complete control (as in easily locating content within a book or library) may not offer this sort of interactivity and thus necessarily no opportunity for an experience of presence. Implicitly, this argues that there must be a second actor or agent, at least partially independent of the human user, so that the user can detect this agency and infer presence.

While research has intentionally varied and developed different levels of interactivity and presence in cancer-related Internet communication venues (to be discussed below), there are a number of other attributes we have identified through observational research that also deserve consideration. Indeed, in hundreds of support groups operating on the Internet ad hoc as self-organizing conversations with no particular oversight or administration, important communication characteristics may offer valuable considerations and modifications of developing communication support systems. Organic Internet discussions, such as Usenet support groups, range from noncancer topics about social situations (eg, alt.support.divorce) to other health-related topics. Among the several cancer-related discussions, participants discuss pharmacological questions and answers, as well as exchange coping and emotional advice. These discussions are surprisingly revealing, with participants often baring their souls with highly intimate narratives. They feature all the categories of traditional social support, such as information, esteem, network, and emotional support; whereas, due to the distributed, electronic nature of the interaction, material support is less frequently arranged via these verbal relationships [25]. A number of characteristics of these online discussions warrant attention as well.

### Homophily

One of the most striking benefits of online support groups is the way they bring out common experience, or homophily, among participants. Perceived similarity is well known to produce feelings of attraction and increase a person's tendency to be persuaded in communication of all kinds. Some of the earlier theories and commonplace assumptions about computer-mediated communication suggest that similarity might be hard to detect online: “As a result of limited nonverbal cues in on-line environments, individuals may find it difficult to assess similarity” [26] (p. 48). However, several factors mitigate this potential problem. First, according to the social identity/deindividuation model of computer-mediated communication [27], it is the social identity, or social similarity of online communicators who have a common life experience, that drives identification and relating in online interaction. Research on the “hyperpersonal model” of computer-mediated communication [23] shows how intense relationships develop through language alone among online cancer support group members over time [28]. Participants in an online support group select the group and know the purpose, and they relate to one another very strongly based on a well-founded and high degree of similarity.

The messages on these systems are often narrative and conversational in form, helping users to relate to common situations and experiences, thereby reinforcing the value of these interactive discussions [29]. In many cases, discovering that there are others going through the same physical and emotional experiences provides a good deal of psychotherapeutic value in and of itself. It is common to see message postings praising the existence of an online venue that has shown a newcomer that there are hundreds of others “just like me.” Finding someone “just like me” is not only possible, it is more probable in a group of hundreds of online cancer patients than among a small circle of close offline friends. Indeed, Wright [26] found a significant empirical relationship between a measure of homophily and support satisfaction in a survey of online support group users.

### Social Distance: Expertise and Stigma Management

Although the homophily principle highlights the benefits of perceived similarity among users of an online cancer discussion, the differences among users and the fact that they do not know one another offline—their “social distance”—adds complementary benefits. Applying sociometric principles to online social support, Walther and Boyd [30] identified some advantages of communicating with strangers in their analysis of the attractions of online support. The first advantage draws on the notion of “the strength of weak ties” [31]. This principle highlights that our common groups of friends and acquaintances—our “strong tie network”—often does not contain people with expertise or familiarity with an issue that might be beneficial to us on a specific issue such as cancer treatments. Indeed, the literature on traditional, face-to-face social support suggests that close friends and family members may become uncomfortable, and are often ineffective, when trying to help patients or other people with problems address their concerns [32]. However, in online discussions, people with different expertise, at different stages of illness or recovery, yet whose experience maps on to support seekers in some way, are available at the click of a mouse. This distributed expertise represents a bona fide advantage to cancer patients looking for advice from online support groups.

The fact that online support providers are not part of support seekers' day-to-day physical lives offers another benefit: the management of stigma and embarrassment. Social support seekers are, by definition, having trouble. Describing the emotional, physical, and social problems they are dealing with often means admitting vulnerability or disclosing potentially embarrassing conditions. In some cases, it would be more embarrassing for one's day-to-day colleagues and friends to be aware of either the problems or of the lack of control implied by needing help [33]. As well, face-to-face friends tend to minimize and downplay the seriousness and distress of individuals who seek support for their problems [32], which, while well intended, is ineffective and may further one's embarrassment. Moreover, discussing breasts or testicles or other “private parts” violates mores in other social contexts. When dealing with groups and individuals whom one knows strictly online, however, and whose existence does not intrude on other social or professional social networks, these negative impacts are ameliorated. There is less reason to hold back and less fear of embarrassment since the confessors are unlikely to run into each other elsewhere or share information with people in other domains of their lives. Things confessed online are unlikely to travel back to the office rumor mill.

### Anonymity and Privacy

This segregation of support sources is further enhanced by another feature of online support—anonymity. Anonymity online comes in several forms. The relative anonymity of interacting online with a set of people who are segregated from regular social partners, as discussed above, is one version. By using email addresses or log-on names that are not immediately traceable to offline identity, social support users may take further advantage of the ability to post personal questions and details of their problems or solutions without having this information connected to their offline lives. The use of a “hotmail.com” address or the deployment of anonymous Internet-based message systems (see [34]) provides various levels of masking the identity of the message sender from the content of the message. In this day and age of traceable, searchable Web archives, the ability to use a pseudonym and be anonymous when exchanging personal information (in a way that is impossible to link the information to the author) is rare and potentially valuable.

In a related vein, online health information systems can create a sense of privacy [35,36] similar to that achieved in interpersonal interactions because of the one-on-one interaction with the computer. Privacy is important for users in order to disclose risky health behavior [37]. It also may be a factor that determines whether individuals will seek information on health problems, particularly those that carry some stigma (eg, HIV/AIDS) or are illegal (eg, smoking by adolescents).

### Interaction Management

Interaction management is a concept reflecting another attribute of online cancer support that is more difficult to capture in offline support dynamics. According to Walther and Boyd [30], interaction management occurs at two levels: the degree of participation a participant wishes to have in an online group, and the way that individuals are able to express themselves when they participate. In online support groups, support seekers may avail themselves of system resources opportunistically, seeking or providing information when the need arises and retreating when their information needs recede. Although reciprocity and presence are important aspects of a vibrant community, online or off, there are times when a participant may be too ill, or too depressed, to wish to witness others' exchanges. Likewise, there are times when individuals are not strong enough to reciprocate the advice they have received, and online support groups allow users to retreat, without contest, when they need to do so. In offline relationships—especially the intimate ones in which social support is exchanged—obligations to reciprocate and aid others may persist, even when it is all one can do to cope with one's own illness or life circumstance.

Interaction management at the level of individual expression refers to the manner in which computer-mediated communication allows us to craft the messages we share with others, in ways that are often uncommon in face-to-face speech. Far from being the cold and empty vessel for communication that early theories and research described online interaction to be, research and experience show that social and emotional presence are real virtues of online groups. Computer-mediated communication allows us to create messages asynchronously, in the absence of our addressees, and provides editing capability. These technological attributes facilitate the purposeful and deliberate choice of words users employ as they describe difficult issues or work to provide sensitive responses. Recent research has documented that, in computer-mediated communication sessions, users take more time and edit messages more when they are addressing an audience that matters to them. They engage greater cognitive resources and make messages friendlier and more sophisticated when attempting to craft impressions on others online [38]. Online communicators are no less effective emotionally when relying on words alone than are counterparts in face-to-face interactions, who have both words and nonverbal cues at their disposal [39]. Indeed, one respondent in Walther and Boyd's study [30] described the communication in online support groups as “a purer form of communication” than face-to-face interaction: “Writing is a lot different means of communicating than we are all used to. Our questions and answers are more articulate, more meaningful, and can be viewed over and over again until we get the message. It is my belief that the discussion is easier and healthier…” (p. 180).

## Outcomes of Internet Communication Attributes

What are the known and suspected effects of variations in the attributes of cancer-related communication systems? Obviously, the ultimate ends will be prevention, better decision making, better health, and coping. In order to achieve these objectives, communication must achieve intermediate-level outcomes such as learning and social influence.

### Learning

The presentational format in online health information programs can affect learning of its content. Recent studies found that user control enhances elaboration and learning of complicated concepts that require understanding linkages between concepts. However, user control also increases selective scanning of online information that can interfere with learning, especially of simple content that mainly requires comprehension and memory [40,41]. To the extent that interactivity produces a sense of mutuality and involvement, source credibility should be enhanced, improving the believability of information conveyed. Thus, interactive interfaces may be most effective when teaching users complicated concepts that require deeper thought and understanding of relationships between information. The delivery of simple straightforward information may be most effectively done with less interactivity, to insure that users learn the information and do not miss it as they scan Web pages and email messages.

### Social Influence

Patient compliance is a problem in medicine and especially when patient lifestyle changes are considered [42]. Explanations for the success of compliance-gaining communication strategies suggest that compliance depends on perceptions of reciprocity, social obligation, and source credibility (built upon a sense of relationship with the source, even in fleeting interchanges) [43-45]. Interactive methods using telephone or interpersonal contact for recruiting patients to health services such as smoking cessation programs are much more successful than passive recruiting methods that rely on mass media or direct mail [46]. Interactivity of online health information services has the potential to create a sense of mutuality, connection, common ground, and shared understanding, and, ultimately, participation in medical decision making [47]. This should heighten positive feelings toward health care providers and increase their credibility and the trust placed in them [48,49] to improve interpersonal influence [50,51]. The credibility of information can also increase as a medium becomes “richer” in sensory channels [52,53], such as when online systems utilize the multimedia features of the World Wide Web. Alternatively, new features related to the Web itself may promote or hinder credibility, such as the top-level domain of a health Web site, and the interaction effects of domain and the presence or absence of advertisements [54]. As noted earlier, online services can create a sense of privacy that may be important for promoting the exchange of information, perceptions of reciprocity and obligation, and ultimately compliance. Recently, one study was able to implement Internet-based recruitment strategies for an online smoking cessation program that were found to be more effective than traditional nonelectronic ones [55]. It is important to note, though, that the increasing amount of unsolicited email or “spam” threatens to reduce the credibility of online information. However, spam may mostly affect the credibility of *unsolicited* online communication. Online communication generated from known individuals or through a process called permission-based marketing—where users agree to receive follow-up information after obtaining services over the Internet—should continue to have the potential to influence [56].

## Two Exemplars

How do these attributes and their intermediary effects combine to affect prevention, decision making, and coping? Two examples are offered. Interactivity has been demonstrated to have valuable direct and indirect effects in different Internet systems related to cancer. We will review its indirect relationship, through its effect on presence, further below. In another case, interactivity in terms of tailoring specific information for different computer users has been shown to have positive effects on smoking prevention and smoking cessation through its enhancement of learning and social influence. Recent innovative uses of computerized and Internet programs to prevent risk behaviors by adolescents have had some success, including Web-based programs to reduce adolescent smoking.

### Interactivity, Learning, and Influence in “Consider This”

An original online tutorial system, Consider This, was developed by one of our authors and his colleagues to be part of school curricula, with the following principles of interactivity in mind: “[to] tailor program content to adolescents' intentions and experiences with smoking to counter desires to try smoking, provide support for not smoking in social contexts with opportunities to smoke, and address experiences with cigarettes that can promote further smoking…. Tailored content is provided through software routines controlled by a backend SQL database…allowing it to be delivered in real time as the person uses the program” [57]. Interactivity and message tailoring were facilitated by having adolescents respond to online questions and by tracking their use of program activities.

The Consider This Web program featured 73 online activities organized into six interactive multimedia modules based existing smoking prevention and cessation programs for youth, as well as other sources. The modules employed a host of interactive activities using audio narration, sound effects, and music in order to engage users' senses, and they featured attractive peer models in order to engage adolescents' attention. The content was “designed to create positive outcome expectancies for not smoking, negative outcome expectations for smoking, and self-efficacy expectations for avoiding or stopping tobacco use” [57]. The activities in the modules provided non-directive counseling with reasons for not smoking, and, employing the interactivity of the system, matched smoking avoidance arguments with core personal values through a motivational interviewing technique.

Consider This was tested in parallel randomized efficacy trials from 2001 to 2002 in the United States and Australia. The study found evidence that Consider This was successful at moving perceived norms and beliefs related to smoking in the desired direction (ie, to be less favorable about smoking). There were differences between the national samples in terms of specific behavioral outcomes, but both samples showed a reduction in intention to smoke—a critical variable in the age group studied—among those who used the program.

### Interactivity, Presence, and Coping in CHESS

For the past 15 years, a subset of our authors has been developing and testing generations of an interactive cancer communication system (ICCS) called CHESS (Comprehensive Health Enhancement Support System). This ICCS is an online system that integrates a range of services that can be described as information (ask an expert, questions and answers, instant library, resource guide, personal stories, Web links), support (online discussion group, ask an expert, personal stories), and skills building (journaling, decision making, action planning, managing distress, healthy relating). Over a series of randomized clinical trials, this ICCS has demonstrated significant improvements in cancer patients' quality of life, especially for underserved audiences [58].

As part of the activities of the Center of Excellence for Cancer Communication Research (funded by the National Cancer Institute), research and development over the last year have been directed toward amplifying a sense of presence in the CHESS system. In the following discussion we review the relationship between presence and interactivity, the methods intended to heighten cancer patients' sense of presence in this specific ICCS, how this sense might mediate effects on quality of life, and how these mediation effects may be measured.

A major strength of this and similar ICCS programs is that they are indeed systems. Whereas most websites provide a single approach to content, forcing a user to browse from site to site to meet different kinds of needs, an integrated system of services meets the varying needs of its users (eg, a breast cancer patient) at different times and in different situations. The systems approach not only makes it far easier for users to find what they need, but it may also encourage them to see connections between physical, emotional, and social aspects of their illness.

CHESS is also interactive in the sense that it maximizes opportunities for user control and allows users to feel that the ICCS is responsive to them [59]. Lee's argument that there is an inextricable link between interactivity and social presence [24] dictates that interactivity is likely a necessary condition for online presence to occur. However, dealing with the relationship between interactivity and presence raises some distinctions within interactivity that must be considered. One current project is attempting to decompose CHESS to determine which kinds of content are responsible for its benefits. From this perspective, despite the depth and quality of CHESS modules during the past decade, and its characterization as a purportedly “interactive” medium, dividing the many services into distinct elements makes it evident that the various components represent three very different kinds of interactivity, which can be understood through the following three metaphors.

- The ICCS as a “book index”: Users control where they go, but the system is not proactive.
- The ICCS as a “telephone”: The system connects human users (via email, bulletin boards, Web logs).
- The ICCS as “coach/collaborator”: The system tracks and remembers the user and responds in accord with that history.

This breakdown makes several conclusions stand out. First, connections to real individuals have been an important part of CHESS from the beginning, but the recognition of the contributions these connections make to social presence and its potential benefits are just becoming clear. Second, new developments and expansions of what were rudimentary capabilities have the opportunity to create a virtual social presence of the CHESS system itself, and new designs are being undertaken with presence explicitly in mind.

A prime example of connection to other real people is CHESS's bulletin-board style Discussion Group, which has always been a central focus for users, often accounting for two-thirds or more of all uses of the system [58]. Drawing on many of the attributes enumerated above, patients report in many ways that it is not merely the additional information that sharing experiences provides that is important about the Discussion Group. Instead, there is a sense of community and social support. In other words, breast cancer patients see the CHESS Discussion Group as providing social presence through connecting them with other real women. Similar reactions occur to Ask an Expert, in which users can write questions that a human expert (usually a Cancer Information Service information specialist) will answer within 24 to 48 hours. Here, the social presence is again in the connection with another real person, but with a professional rather than a peer.

Social presence should also increase as CHESS expands coaching and adds collaborating to its services. Implementations such as Action Plan and Decision Aid have always provided guidance for users making decisions or attempting behavior change. But the construction of additional modules, such as Managing Distress and Healthy Relating, adds the tools for much more assessment and feedback, based both on users' response choices and on their individual situations and perceptions. That is, to effectively “coach” a patient who is developing and beginning to employ new skills, the system will provide example situations and evaluate patient response choices. Although there is no human behind the machine in this case, this clearly still meets the criterion of interactivity through interdependent exchange of information since the patient gets feedback and guidance from the system.

The “collaborator” role of tailoring the system to the patient is a fresh addition to CHESS. Whereas tailoring attempts such as Consider This and others deliver the most relevant and beneficial message to a user [60], such an approach is not appropriate for a large system of information, support, and tools designed to be used repeatedly over time. As things change over time, the appropriate message must change too. As in all tailoring, CHESS assesses the user's situation and status, and then the system uses that information to help the user get to the content that will be most relevant and beneficial.

#### Future CHESS Research

It would be unfair to present the initial CHESS system as a full-fledged expert system, but the constraints and commonalities of the breast cancer situation offer the opportunity to do a great deal with relatively simple algorithms. For example, knowing the calendar of a woman's treatment plan (obtained from the medical record at recruitment and alterable by the user at any time) allows us to present a narrow set of treatment tips that match what the woman is experiencing, or will shortly experience. Beyond this, she is encouraged to report her current emotional and functional status and concerns, which further allows the system to recommend a narrower version of CHESS content that is better suited to her. To keep this functioning, her personal home page contains a link (“What CHESS knows/assumes about you”) so that she can review and alter this at any time. She can also elect to turn off tailoring and use the system in “index” mode. And as with coaching, these collaborations should provide considerable virtual social presence.

However, beyond connection to real others and the virtual presence of a coach/collaborator, investigation of social presence within CHESS has revealed other potentially fruitful avenues. It is possible that even an effective Google search can create a sense of presence; the AskJeeves search engine, which shows what queries other users have recently made, seems designed to do just that. If search engine sites can create presence, we need to reconsider the nature of agency as a necessary condition. Perhaps the social presence some people experience from Google stems from its typical performance of providing both highly appropriate links and some surprise or unpredictability in what it returns. Alternatively, highly experienced Google users probably understand its algorithm and may be finding presence in the feeling that its results provide a sense of collective behavior of many Web users.

Attention should focus on the combination of two attributes—appropriateness and unpredictability of response. A “book index” type of ICCS takes the user directly to highly appropriate but very predictable content. Other humans posting to discussion groups provide appropriate (though variable) responses to the user, but with some degree of unpredictability that is characteristic of independent agency. Programming-based coaching or collaborating can potentially be both highly appropriate and unpredictable, though achieving this is difficult and errors can be costly.

#### Perceptions and Mediation

For the most part, breast cancer patients are likely to experience CHESS's social presence because of the Discussion Group's ability to connect them with other women, the coaching of skill-training components, and the collaboration of tailoring CHESS to their situation. Based on the following assumptions, several hypotheses can be articulated regarding the kinds of perceptions that will then mediate greater CHESS effects:

- The Discussion Group, especially, should produce a sense of community with shared experiences.
- A variety (or combination) of CHESS interactive components should provide some sense that the patient is being watched over and protected, no matter whether it is a group of real women who are keeping track of her or a computer coach/collaborator.
- With Ask an Expert as well as the computer coach/collaborator, this protection comes with the additional perception of expert reliability and power. However, for some patients, support from fellow cancer patients is particularly powerful because of the expertise of having been or currently being cancer patients themselves [61,62].

These perceptions should lead to several mediating effects that will then lead to an increase in the degree to which CHESS affects such things as emotional well-being, functional well-being, information competence, and effective interaction with health care providers. Hypothetically, all these perceptions, especially if they are enhanced by perceived expertise, should buffer negative affect. This is important because negative affect can be debilitating and can shut off effective coping behaviors. Also, the encouragement and support provided should bolster self-efficacy, the sense that the individual is capable of effective actions. Further, guidance from the collaborator should focus patients' use of CHESS on more effective varieties of use [63]. For example, use of Discussion Group appears to be more beneficial if combined with the use of other kinds of CHESS services or if the user is an active contributor instead of just “lurking” and reading messages [64]. Finally, by providing patients more individually relevant information and tools, the perceived utility of CHESS content should be greatly enhanced overall, which should increase system “stickiness.” In past studies, substantial proportions of patients have used CHESS for only a few weeks and then discontinued use. Some of them may well have gotten all they needed from the system. Others probably would have benefited from returning as their situations changed (eg, as treatment continued or ended), and greater stickiness should enhance this.

## Caveats

The preceding review has focused on structural system and social characteristics of several types of interactive online health information systems and has discussed the potential benefits of various combinations among them. While this review has focused on characteristics of the online modality, it is important to recognize that communicators often effectively compensate for structural shortfalls if given adequate time and motivation [23,65] and adapt technology to existing communication practice [66-68]. The combination of communication outcomes, modality features, and audience characteristics will determine the success of Internet health information programs.

Clearly, a bias throughout much of the above has been that social presence is desirable and that ICCS designers should enable users to perceive it as much as possible. In part, this results from the perception that current ICCS users are likely to experience relatively little social presence, so that increasing it would clearly be a step in the right direction.

Nonetheless, we must recognize that social presence is not automatically desirable here or in other computer-based health enhancement systems. Patients may regard the social presence as an unwelcome “big brother” who knows too much about them or is being too intrusive. And errors (responding inappropriately to user) could undermine system credibility or produce boomerang effects.

The response so far has been to push forward, but with several safeguards. First, the CHESS project is pilot testing the tailoring mechanisms in paper prototype and pilot versions with prior CHESS users to try to establish what levels of system activity stimulate presence perceptions without producing negative reactions. And, second, even when new additions to the system roll out, plans call for users to be allowed to turn off or avoid these features at their own discretion.

Another final caveat is raised by the emerging problem of low return use or drop off in use of online health information systems. Many of the programs evaluated recently depended upon the user to initiate contact and “pull” information from them, and there was no guarantee that the at-risk population would use them just because they were available, even when assigned to do so [1,7]. Low use can reduce the effectiveness of Internet health information systems [6,7,10,69]. There is scant information on the factors that improve website use; use may be higher among young users, those recently diagnosed with a disease, and users expressing intentions to change or who are actually making a change [70]. Some advertising researchers have speculated that interactivity of these systems increases return visits [71]. Recently, a few researchers have observed that email notifications (a crude form of interactivity) increased use of Internet health programs [9,10,72].

## Conclusions

Continued study of the efficacy of online health information systems is essential because they are expensive to create and governmental and non-governmental health organizations are quickly embracing them. Different levels of access to the Internet can present barriers to the production and delivery of these systems [69,73]. Fortunately, many of the disparities in Internet access based on gender, race, and socioeconomic circumstances have shrunk substantially in the United States: Internet access is nearly universal in schools [74] and is present in over half of US households [75]. Government and nongovernmental organizations that seek to deliver health information must have a good understanding of how to deploy the features of online health information systems most effectively, about which, unfortunately, current knowledge is limited. There is a risk that health professionals will become disenchanted with these Internet health information systems unless researchers test how the features affect important outcomes that determine the health of populations.

## Abbreviations

CHESS: Comprehensive Health Enhancement Support System
ICCS: interactive cancer communication system
